# A composite activity-sleep index is associated with metabolic syndrome severity in adults with diabetes

**DOI:** 10.1007/s40200-026-02072-6

**Published:** 2026-09-23

**Authors:** Samuel Akyirem, Emmanuel Ekpor, Eric Peprah Osei

**Affiliations:** 1 College of Nursing, University of Kentucky, Lexington, KY USA; 2 The Australian Centre for Behavioural Research in Diabetes (ACBRD), Diabetes Victoria, Carlton, VIC Australia; 3 School of Psychology, Deakin University, Geelong, VIC Australia; 4 College of Nursing, University of Illinois Chicago, Chicago, IL USA

**Keywords:** Metabolic syndrome, activity-sleep index, 24-hour movement behavior, Secondary data analysis

## Abstract

**Purpose:**

To investigate the association between Activity Sleep Index (ASI) and Metabolic Syndrome Severity (MSS) scores in a nationally representative sample of U.S. adults with diabetes.

**Methods:**

We conducted an analysis of the 2011-12 and 2013-14 cycles of the National Health and Nutrition Examination Survey (NHANES). MSS scores were calculated using sex, systolic blood pressure, diastolic blood pressure, triglycerides, high-density lipoprotein cholesterol, blood glucose, and waist circumference. A higher MSS was indicative of worse cardiometabolic health. ASI was calculated based on average time spent in moderate-to-vigorous physical activity (MVPA), light-intensity physical activity (LIPA), sedentary behavior, and sleep derived from raw accelerometer data. Multivariable linear regression was used to assess the association between ASI and MSS.

**Results:**

Overall, 437 adults with diabetes were included in the current analysis. The mean age of participants was 60.7 years. A majority of participants had obesity (60.5%) but only 27.1% had a history of at least one cardiovascular event. Higher ASI z-score was significantly associated with lower MSS after adjusting for all relevant covariates (B=-0.44, 95% CI: -0.70, -0.18). We also found that current use of insulin and oral antihyperglycemic medications as well as never-married status were associated with significantly higher MSS. In contrast, increasing age was associated with lower MSS.

**Conclusion:**

Healthier physical activity and sleep patterns were associated with lower cardiometabolic risk among adults with diabetes.

**Clinical trial number:**

Not applicable.

**Supplementary Information:**

The online version contains supplementary material available at https://doi.org/10.1007/s40200-026-02072-6.

## Introduction

Diabetes affects 12% of individuals living in the U.S. [1]. In 2023, diabetes was reported as a contributing cause in more than 300,000 deaths in the country [1]. Individuals living with diabetes are at increased risk for cardiovascular complications, poor health-related quality of life, and all-cause mortality [2]. Physical activity and sleep are essential aspects of diabetes self-management, with evidence suggesting that these behaviors are independently associated with better diabetes-related health outcomes [3, 4]. While many studies examine the impact of these behaviors on health outcomes in isolation, there is a growing consensus regarding the need to consider physical activity and sleep concurrently to determine their combined association with health outcomes [5, 6].

Several approaches exist to analyze the collective impact of physical activity and sleep on health outcomes in adults with diabetes. Previous studies have used latent profile analysis (LPA) to identify classes of individuals based on their movement profiles, isotemporal substitution analysis (ISA) to identify changes in health outcomes when time is reallocated from one behavior to the other, and compositional data analysis (CoDA) to model the relative distribution of time spent in each movement behavior in a day [6, 7]. Although these analytic approaches allow researchers to consider multiple movement behaviors concurrently, they are not designed to generate a single integrated metric that captures overall physical activity and sleep patterns. To address this gap, the Activity-Sleep Index (ASI) was developed as a composite score that integrates moderate-to-vigorous physical activity (MVPA), light-intensity physical activity (LIPA), sedentary behavior, and sleep into a single metric [8]. The original ASI incorporated multiple dimensions of sleep and physical activity, including sleep quality and daytime alertness [8]. More recent versions have used a more parsimonious set of indicators focusing on time spent engaging in each behavior thus, demonstrating the flexibility of ASI as a measure that can be adapted to available data while still capturing overall movement behavior patterns [9, 10]. In the general population, higher ASI scores have been associated with lower risk of depression, anxiety, and stress [8]. However, ASI has not been examined in adults with diabetes, a population in whom movement behaviors are generally linked to cardiometabolic health [11]. It is, therefore, unknown whether ASI is associated with cardiometabolic risk in adults with diabetes.

In adults with diabetes, metabolic syndrome severity (MSS) score is commonly used to capture cardiometabolic risk [12, 13]. MSS has been shown to predict adverse cardiovascular outcomes and mortality in adults with diabetes [14]. MSS provides a more nuanced assessment of cardiometabolic risk than dichotomous definitions of metabolic syndrome by capturing the continuum of metabolic dysfunction [13]. Given that both sleep and physical activity are key behavioral determinants of metabolic health [4], a composite ASI may offer a valuable framework for understanding how integrated movement behaviors relate to metabolic risk in this population. Therefore, the purpose of the present study was to investigate the association between ASI and MSS in a nationally representative sample of U.S. adults with diabetes. We hypothesized that higher ASI scores, reflecting healthier or favorable combined activity and sleep patterns, would be associated with lower MSS. Establishing an association between ASI and MSS would provide preliminary evidence supporting the utility of ASI as an integrated behavioral metric and a potentially clinically relevant outcome for future interventions that simultaneously target sleep and physical activity (including sedentary behavior) in adults with diabetes.

## Methods

### Study design

This study was a secondary analysis of cross-sectional data from the 2011–2012 and 2013–2014 cycles of the National Health and Nutrition Examination Survey (NHANES). NHANES is an annual study conducted by the US Center for Disease Control and Prevention (CDC) to obtain national-level estimates of the health status and patterns of health behaviors of individuals living in the US. The study includes both survey and examination sections. The survey section includes closed-ended interviews guided by questionnaires. The examination portion is carried out by Mobile Examination Centers (MEC) and includes laboratory tests, physical assessments (e.g. body mass index [BMI]). NHANES uses survey weights to account for the complex survey design and survey non-response. Survey weights ensure that findings are generalizable to noninstitutionalized civilians living in the US [15].

### Selection of study participants

**Fig. 1:**
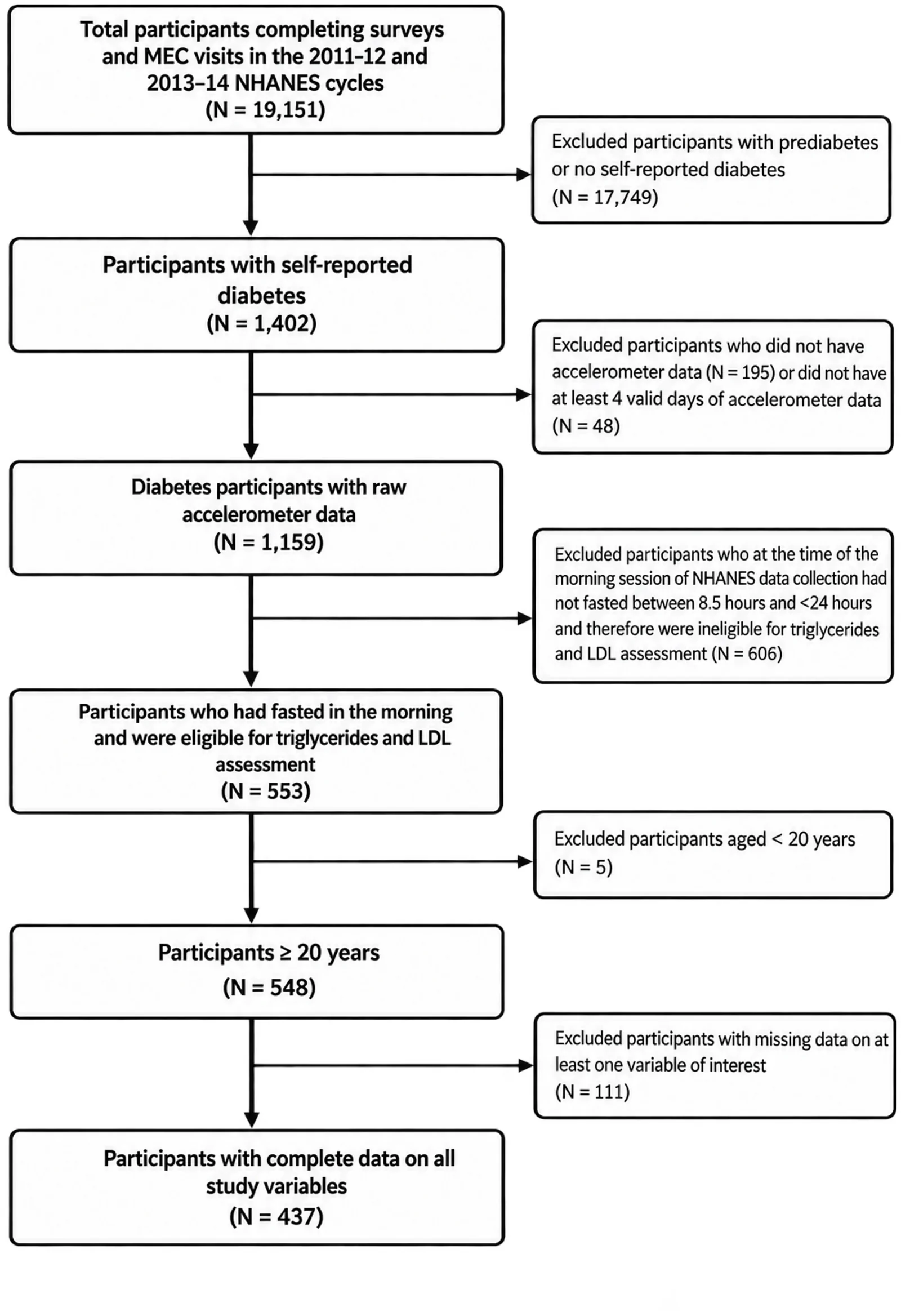
A flow chart showing how the final analytic sample was selected

In the current analysis, we included adults aged ≥ 20 years with diabetes (determined as responding “yes” to the question “have you ever been told by a health professional that you have diabetes?”), had raw actigraphy data, and had complete data on all other variables of interest. Overall, 1,402 participants with self-reported diabetes completed the interviews and MEC components of the 2011-14 cycles of NHANES. Participants who did not have raw accelerometer data (*N* = 195) or did not have at least 4 valid days of accelerometer data (*N* = 48) were excluded. We also excluded participants who did not meet NHANES fasting requirements (8.5 to < 24 h of fasting before the morning examination session) and were therefore not eligible for triglyceride and LDL cholesterol assessment (*N* = 606). After excluding those who were aged under 20 years (*N* = 5) and those with missing data on at least one variable (*N* = 111), 437 adults with self-reported diabetes remained, which formed the final analytic sample, as shown Fig. 1.

### Composite activity-sleep index

Individuals aged 3 years or older and participating in the 2011–2014 NHANES cycles were invited to continuously wear a physical activity monitor, ActiGraph GT3X+ (ActiGraph, Pensacola, FL), for seven full consecutive days starting on the day of their exam in the NHANES MEC, to objectively assess physical activity and sleep patterns [16]. The device was placed on a mesh wristband and worn on the non-dominant hand as appropriate. Participants were instructed not to do anything with the device except to wear it. If the participant needed to remove it, for any reason, they were asked to put it back on the same wrist in the same orientation, as soon as possible [17]. The device recorded accelerometer data at a sampling rate of 80 Hz resulting in 288,000 records per full hour. Additional details on how accelerometer data were collected in NHANES are reported elsewhere [17].

Raw accelerometer data in .csv format were obtained for each participant for further processing. We used the GGIR package in R for data processing [18]. First, GGIR performed auto-calibration for each file, comparing non-movement periods to the gravitational acceleration to adjust for any calibration errors. GGIR then computed the acceleration metric Euclidean Norm Minus One with negative values rounded to zero (ENMO) over a 5-second epoch. Sleep period time (SPT) window was identified using the Heuristic algorithm looking at the Distribution of Change in Z-Angle (HDCZA algorithm) [19]. A calendar day was defined from midnight to midnight and considered valid if it contained at least 16 h of recorded data. We only included participants with at least 4 days of valid data. Sedentary behavior, LIPA, and MVPA were classified using recommended acceleration thresholds of < 44.8 mg, 44.8–100.6 mg, and > 100.6 mg per epoch, respectively [20]. Specific details of how the GGIR package was set up can be found in the “config.csv” file (supplementary file S1). For all subsequent analyses, we used the Part 5 person-level summary report generated by the GGIR package, which provides weighted average (5:2 weekdays to weekends) minutes spent in MVPA, LIPA, sedentary behavior, and sleep per day.

We then derived activity sleep index (ASI) as a composite score to summarize all four movement behaviors (sleep, MVPA, LIPA, and sedentary behavior). ASI was chosen because it incorporates multiple movement behavior dimensions into a single summary score that can be readily computed within datasets and provides an integrated measure of overall activity-sleep patterns. ASI was created by first rescaling each movement behavior time from 0 to 10 using the formula:

$$\:Rescaled\:movement\:behavior=10\:\times\:\:\frac{X-{X}_{\mathrm{m}\mathrm{i}\mathrm{n}}}{{X}_{range}}$$

Where X is the observed amount of time spent in a movement behavior, $\:{X}_{min}$ is the minimum observed time spent in the movement behavior and $\:{X}_{range}$ is the difference between the minimum and maximum time spent in the movement behavior. Sedentary time scores were reversed such that higher scores indicate less sedentary time and more favorable behavior. Rescaled items were then summed up to produce a total ASI that ranges from 0 to 40 with higher scores indicating a lower risk or more favorable movement behavior pattern.

### Metabolic syndrome severity score

Metabolic syndrome severity (MSS) scores were calculated using the following variables: sex (male vs. female), systolic blood pressure in mm Hg, diastolic blood pressure in mm Hg, triglycerides in mmol/L, high-density lipoprotein cholesterol (HDL) in mmol/L, blood glucose in mmol/L, and waist circumference in centimeters. Details of how these data were collected have been previously reported [21]. The R package, *pscore*, uses the above variables to compute MSS scores. Specific details of how *pscore* calculates MSS scores are published elsewhere [22]. The *pscore* package uses principal component analysis to calculate the multivariate distance of an individual’s cardiometabolic risk profile from sex-specific clinical thresholds rather than from the sample mean [22]. While MSS was initially developed in the general population, evidence shows that the score can also be reliably generated using participants who are currently taking medications for chronic conditions, including diabetes (*r* = 0.98) [22]. A higher MSS indicates worse cardiometabolic health.

### Sociodemographic and clinical variables

The following sociodemographic and clinical variables were included in the study: age (continuous), gender (male/female), race and ethnicity (recoded as Non-Hispanic White/Non-Hispanic Black/Hispanic/Others), education (recoded as up to high school/some college/college graduate or higher), marital status (recoded as never married/married or living with partner/separated or divorced or widowed), BMI (normal [18.5–24.9 kg/m^2^], overweight [25.0–29.9 kg/m^2^], and obesity [≥ 30 kg/m^2^]), poverty-income ratio (recoded as below federal poverty level [FPL]/100–200% above FPL/>200% above FPL), smoking at least 100 cigarettes in life (yes/no), current use of insulin (yes/no), current use of oral antihyperglycemic medications (yes/no), and adverse cardiovascular events (yes/no to a history of stroke, heart attack, heart failure, coronary artery disease, and angina pectoris). These variables were used to describe participants’ characteristics, and selected variables were included as covariates in the regression model, as described in the Statistical Analysis section.

### Statistical analysis

Statistical analysis was conducted with R version 4.3.0. Survey weights (*WTSAF2YR;* fasting subsample weight) were applied to account for the complex survey design of the NHANES and to generate estimates that are representative of all U.S. non-institutionalized adults [15]. We divided *WTSAF2YR* by two because we combined two NHANES cycles. We computed weighted descriptive statistics using means, standard deviations, counts, and proportions of study variables. Multivariable linear regression was used to model the association between ASI and MSS while adjusting for age, poverty-income ratio, marital status, smoking status, adverse cardiovascular events, education, current use of insulin, current use of oral antihyperglycemic medications, and race and ethnicity. For the regression analysis, ASI was converted to z-scores to aid the interpretation of its regression coefficient. We report regression estimates and their corresponding 95% confidence intervals. A significance level of 0.05 was chosen for all inferential analyses. Regression diagnostics were conducted using design-adjusted Pearson residuals to account for the complex sample design. Visual inspection of weighted residual plots confirmed that the assumptions of linearity and homoscedasticity were not clearly violated. An additional model including a quadratic term for ASI showed no evidence of nonlinearity, as the quadratic term was not statistically significant (*p* = 0.817). A survey-weighted Q-Q plot also demonstrated a mild, light-tailed error distribution. Multicollinearity was minimal; all intercept-adjusted survey variance inflation factors (VIF) were below standard thresholds of 5, with a maximum VIF of 1.46 for the “college or more” dummy variable (computed with “svyvif” function in the R package “svydiags” [23]). Missing data were handled through listwise deletion, resulting in only complete-case analysis. There was no significant difference in the marital status, education level, race and ethnicity, income level, smoking status, age, ASI, MSS, and cardiovascular disease between participants included in the final sample (n = 437) and those excluded due to missingness (n = 111) (supplementary file S2). Although participants excluded for not meeting the fasting requirement (n = 606) were not directly compared with the analytic sample, the use of NHANES fasting subsample weights accounts for the fasting subsample design and supports population-representative estimates among fasting-eligible participants. Lastly, because MSS includes blood pressure and lipid-related components, we considered whether antihypertensive and lipid-lowering medication use could influence MSS. However, due to missing data for current antihypertensive use (BPQ050A) and current lipid-lowering medication use (BPQ100D), these variables were evaluated only in a sensitivity analysis (N = 207) rather than included in the primary model.

### Findings

Overall, 437 adults with diabetes were included in the analysis. As shown in Table 1, most participants were female (52.5%), Non-Hispanic White (62.1%), married or living with partner (66.5%), and were > 200% above federal poverty level (55.8%). Participants were, on average, 60.7 years old and had lived with diabetes diagnosis for an average of 11.6 years. A majority of participants had obesity (60.5%) but only 27.1% had a history of at least one cardiovascular event. The mean MSS was 4.5. The median time participants spent in sleep, MVPA, sedentary behavior, and LIPA were 405.88, 55.30, 773.68, and 180.61 min respectively (Table 2).

**Table 1:** Characteristics of study participants (*N* = 437)

Variables	*N*	Weighted % (95% CI)
Gender		
Male	220	47.5% (42.2, 52.8)
Female	217	52.5% (47.2, 57.8)
Race and ethnicity		
NH Black	118	14.5% (10.0, 19.0)
NH White	170	62.1% (56.3, 67.9)
Hispanic	101	14.6% (9.8, 19.5)
Others	48	8.8% (5.0, 12.6)
Income level		
Below FPL	112	18% (12.4, 23.6)
100–200% FPL	129	26.2% (19.2, 33.1)
> 200 FPL	196	55.8% (48.1, 63.5)
Marital status		
Married or partnered	268	66.5% (59.8, 73.2)
Separated or divorced or widowed	132	25.1% (20.1, 30.0)
Never married	37	8.4% (4.5, 12.4)
Education level		
Up to HS	232	47.5% (39.6, 55.4)
Some college	126	32.7% (26.8, 38.5)
College or above	79	19.8% (14.1, 25.5)
Smoked at least 100 cigarettes in lifetime		
Yes	213	47.1% (41.2, 53)
No	224	52.9% (47, 58.8)
Positive history of adverse cardiovascular event	115	27.1% (21.8, 32.4)
BMI categories		
Normal	59	10.7% (7.4, 14.0)
Overweight	129	28.8% (22.9, 34.7)
Obese	249	60.5% (54.1, 66.9)
Current insulin use		
Yes	143	31.2% (25.8, 36.6)
No	294	68.8% (63.4, 74.2)
Current oral antihyperglycemic use		
Yes	306	72.6% (66.0, 79.1)
No	131	27.4% (20.9, 34.0)
	Weighted mean	SE
Age	60.7	0.8
Time since diabetes diagnosis	11.6	0.6
ASI (weighted mean, SD)	13.8	4.6
MSS (weighted mean, SD)	4.5	1.7

*CI Confidence Interval, *FPL*  Federal Poverty Level, *HS* High School, *CVD* cardiovascular disease, *ASI* Activity-Sleep Index, *MSS*  Metabolic Syndrome Severity score, *BMI*  Body mass index

**Table 2:** Descriptive statistics of ASI and MSS component variables

	Minimum	Maximum	Median	Interquartile range
ASI components				
Sleep	85.68	798.19	405.88	342.36–473.26
MVPA	0.25	461.42	55.30	26.79–98.57
Sedentary behavior	209.88	1219.93	773.68	672.75–875.08
LIPA	7.87	635.30	180.61	137.89–245.68
MSS components				
Systolic blood pressure	89.33	234.67	128.67	116.00–140.67
Triglycerides	0.35	47.79	1.39	0.98–2.08
Blood glucose	2.17	22.48	7.61	6.27–9.88
HDL-cholesterol	0.49	3.23	1.19	1.03–1.45
Waist circumference	73.9	176.0	106.8	98.3–118.0
Diastolic blood pressure	12.67	98.67	70.00	62.00–76.00

### Association between ASI and metabolic syndrome severity

Table 3 shows the results of the multivariable linear regression. One standard deviation increase in ASI was associated with 0.44-point decrease in MSS after adjusting for all relevant covariates (B=-0.44, 95% CI: -0.70, -0.18). We also found that increasing age was associated with lower MSS (B=-0.04; 95% CI: -0.05, -0.02). Additionally, current use of insulin (B = 0.62; 95% CI: 0.16, 1.08) and oral antihyperglycemic medications (B = 0.79; 95% CI: 0.19, 1.38), as well as never married (B = 0.88; 95% CI: 0.12, 1.63), were associated with higher MSS. The model explained 14% of the variance in MSS.

**Table 3:** Association between activity-sleep index and metabolic syndrome severity score

Variables	Adjusted B (95% CI)	*p*-value
Activity-sleep index (ASI) z-score	-0.44 (-0.70, -0.18)	0.002
Race and ethnicity		
NH White	[Reference]	
NH Black	-0.53 (-1.09, 0.03)	0.061
Hispanic	-0.11 (-0.55, 0.32)	0.596
Others	-0.60 (-1.25, 0.05)	0.069
Income level		
> 200% FPL	[Reference]	
Below FPL	-0.26 (-0.92, 0.40)	0.419
100–200% FPL	-0.15 (-0.49, 0.20)	0.387
Marital status		
Married or partnered	[Reference]	
Separated or divorced or widowed	0.25 (-0.17, 0.67)	0.221
Never married	0.88 (0.12, 1.63)	0.025
Education level		
Up to HS	[Reference]	
Some college	0.23 (-0.22, 0.69)	0.292
College or above	-0.51 (-1.05, 0.03)	0.063
Smoked at least 100 cigarettes in lifetime		
No	[Reference]	
Yes	-0.16 (-0.66, 0.34)	0.511
History of adverse cardiovascular event		
No	[Reference]	
Yes	0.26 (-0.15, 0.68)	0.202
Current insulin use		
No	[Reference]	
Yes	0.62 (0.16, 1.08)	0.011
Current oral antihyperglycemic use		
No		
Yes	0.79 (0.19, 1.38)	0.013
Age	-0.04 (-0.05, -0.02)	< 0.001

Model *R*^*2*^ = 0.14; *p-value* = 0.009

### Sensitivity analysis

Supplementary file S3 provides details of the regression analysis that adjusted for lipid-lowering and antihypertensive medications. The conclusion regarding the association between ASI and MSS (B=-0.39, 95% CI: -0.78 to -0.001) was consistent with the primary model.

## Discussion

In this study, we tested the hypothesis that higher ASI scores, reflecting a more favorable or healthy integrated physical activity and sleep pattern, would be associated with lower MSS. The findings from this study support our hypothesis, showing a significant inverse association between ASI and MSS such that the higher the ASI, the lower the cardiometabolic risk. To our knowledge, this is the first study to determine if ASI is associated with health outcomes in adults with diabetes. The findings highlight the importance of considering physical activity and sleep concurrently in diabetes care and research.

The findings from this study are consistent with previous studies that have used compositional data analysis framework and isotemporal substitution analysis to investigate the association between movement behaviors and cardiometabolic health. For instance, a cross-sectional study of 1549 adults with type 2 diabetes in the Netherlands found that reallocating 20 min a day from sedentary behavior to MVPA was associated with a decrease in waist circumference, HDL-cholesterol, triglycerides [24]. A similar study among 52 adults with type 2 diabetes found that reallocating 30 min from any movement behavior to MVPA was associated with a significant increase in HDL-cholesterol [11]. Additionally, Bogaert and colleagues reported a decrease in waist circumference when time is reallocated from sedentary behavior to sleep [11]. In the current study, we combined multiple indicators of cardiometabolic health into a single continuous metric (i.e., MSS), thereby providing a more holistic assessment of cardiometabolic risk and extending prior findings beyond individual metabolic markers.

Several mechanisms may explain the observed inverse association between ASI and MSS. Higher ASI reflects a behavioral profile characterized by greater physical activity, reduced sedentary time, and healthier sleep patterns, all of which are behaviors that are independently associated with improved metabolic function [3, 4]. Regular physical activity improves insulin sensitivity, supports glucose regulation and improves lipid metabolism [25]. Reduced sedentary time has similarly been associated with better glycemic control and lower cardiometabolic risk independent of engagement in physical activity [26]. Adequate sleep is also critical for metabolic regulation, as both insufficient and poor-quality sleep have been linked to insulin resistance and increased cardiovascular risk [4]. Because MSS incorporates markers such as blood pressure, blood glucose, triglycerides, HDL cholesterol, and waist circumference, healthier or favorable integrated movement behaviors may influence MSS through multiple physiologic pathways simultaneously. ASI may therefore capture the cumulative metabolic benefit of favorable movement behavior patterns.

The inverse association between increasing age and MSS, while not the focus of the current analysis, was unexpected. Contrary to our findings, current evidence suggests that older age is associated with higher MSS among the general population [22]. The association observed in the current study may reflect treatment-related control of metabolic risk factors or characteristics specific to adults with diabetes. It is also important to note that relatively few studies have examined factors associated with MSS among adults with diabetes. Thus, direct comparison of the present findings with prior diabetes-specific studies is limited, and this finding warrants further investigation.

An important implication of this study is the potential utility of ASI as an outcome measure in future interventions targeting physical activity (including sedentary behavior) and sleep simultaneously in adults with diabetes. Most behavioral interventions in diabetes focus on single domains, such as increasing physical activity or improving sleep, despite evidence that movement behaviors are interdependent and compositional in nature such that changes in time spent in one behavior have direct influence on time spent in another behavior [5]. ASI provides a practical way to quantify overall movement behavior health using a single score that reflects multiple behavioral domains. This may make it useful for intervention studies aiming to improve overall movement behavior patterns rather than isolated behaviors. However, to our knowledge, no study has evaluated whether ASI predicts clinically relevant outcomes in adults with diabetes better than alternative approaches such as isometric log-ratios. Therefore, the incremental value of ASI as a summary composite score remains unclear and warrants further investigation.

This study has several strengths. First, we used nationally representative NHANES data, enhancing the generalizability of our findings to noninstitutionalized US adults with diabetes in the fasting subsample. However, exclusions due to incomplete accelerometry or missing covariate data should be considered when interpreting generalizability. Second, physical activity and sleep were measured objectively using wrist-worn accelerometry rather than self-report, reducing recall and social desirability bias. Third, we used MSS, a continuous and clinically meaningful measure of cardiometabolic risk that captures metabolic dysfunction on a continuum rather than relying on dichotomous classification. Several limitations should also be acknowledged. First, the cross-sectional nature of NHANES prevents causal inference. Although higher ASI was associated with lower MSS, it cannot be determined whether healthier movement behaviors led to lower metabolic syndrome severity or whether individuals with better cardiometabolic health were more likely to engage in healthier movement behavior patterns. Longitudinal studies are needed to establish temporal relationships. Second, diabetes status was based on self-report, which may introduce misclassification. Third, because ASI was derived using sample-specific min-max scaling, the absolute ASI score is dependent on the distribution of movement behaviors within the study population. Therefore, ASI values should be interpreted relative to the analytic sample, and future research should evaluate externally derived scaling approaches to facilitate comparisons across studies. Fourth, ASI assumes a monotonic relationship between sleep duration and cardiometabolic health, whereas evidence suggests a U-shaped association [4]. Future refinements of the ASI should also consider scoring methods that assign the highest values to recommended sleep durations while penalizing both short and long sleep. Fifth, the four movement behaviors included in the ASI are compositional, collectively accounting for approximately 24 h of the day. ASI does not explicitly model these interdependencies and should not be interpreted as a compositional measure. Instead, it functions as a summary indicator of overall movement behavior patterns. Additionally, equal weighting was assigned to all four behaviors after rescaling them to a common metric. While this prevents behaviors with larger numerical ranges from dominating the score, the chosen weighting scheme is somewhat arbitrary, and alternative weighting approaches may yield different results. Sixth, although antihypertensive and lipid-lowering medication use was not included in the primary model because of missing data, sensitivity analyses adjusting for these medications produced findings consistent with the primary analysis. Nevertheless, residual confounding by medication use cannot be ruled out because information on medication dosage and duration of use was unavailable. Finally, ASI captures time spent in broad movement behavior categories but does not account for potentially important dimensions such as sleep quality, timing of activity, or patterns of sedentary interruption, which may also influence metabolic health.

## Supplementary Information

Below is the link to the electronic supplementary material.

Supplementary Material 1

Supplementary Material 2

Supplementary Material 3

## Data Availability

All data supporting this study is publicly available on the CDC website at https://www.cdc.gov/nchs/nhanes/index.html.
