# Supplementary material for: A composite activity-sleep index is associated with metabolic syndrome severity in adults with diabetes
Source: J Diabetes Metab Disord. 2026 Sep 23;25(2):264. doi: 10.1007/s40200-026-02072-6 (PMC13601456; doi:10.1007/s40200-026-02072-6)
Supplement: Supplementary file 1 [file 40200_2026_2072_MOESM2_ESM.docx]

**Supplementary file S2. Characteristics of participants included vs excluded from the final analytic sample**

|  | **Weighted %** | | **P-value from Chi-square with Rao & Scott adjustment** |
| --- | --- | --- | --- |
| **Variable** | **Included for having complete data (437)** | **Excluded due to missingness (N=111)** |  |
| Gender |  |  | 0.688 |
| Male | 47.54 | 50.71 |  |
| Female | 52.46 | 49.29 |  |
| Race and ethnicity |  |  | 0.220 |
| NH Black | 14.49 | 21.31 |  |
| NH White | 62.06 | 58.24 |  |
| Hispanic | 14.64 | 6.81 |  |
| Others | 8.81 | 13.64 |  |
| Income level |  |  | 0.454 |
| Below FPL | 18.02 | 27.27 |  |
| 100-200% FPL | 26.15 | 24.37 |  |
| >200 FPL | 55.83 | 48.36 |  |
| Marital status |  |  | 0.415 |
| Married or partnered | 66.52 | 58.33 |  |
| Separated or divorced or widowed | 25.06 | 35.49 |  |
| Never married | 8.42 | 6.18 |  |
| Education level |  |  | 0.108 |
| Up to HS | 47.51 | 59.51 |  |
| Some college | 32.68 | 32.89 |  |
| College or above | 19.81 | 8.28 |  |
| Smoked at least 100 cigarettes in lifetime |  |  | 0.864 |
| Yes | 47.13 | 48.33 |  |
| No | 52.87 | 51.67 |  |
| Positive history of adverse cardiovascular event | 27.06 | 33.97 | 0.352 |
| Age (weighted mean) | 60.67 | 62.27 | 0.601^#^ |
| MSS (weighted mean) | 4.49 | 4.04 | 0.082^#^ |
| ASI (weighted mean) | 13.76 | 12.04 | 0.132^#^ |

ASI = activity sleep index; MSS = metabolic syndrome severity score

^#^ p-value obtained from a weighted t-test
