# Supplementary material for: A composite activity-sleep index is associated with metabolic syndrome severity in adults with diabetes
Source: J Diabetes Metab Disord. 2026 Sep 23;25(2):264. doi: 10.1007/s40200-026-02072-6 (PMC13601456; doi:10.1007/s40200-026-02072-6)
Supplement: Supplementary file 2 [file 40200_2026_2072_MOESM3_ESM.docx]

**Supplementary S3. Results of the survey-weighted model that includes anti-hypertensives and anti-lipid medications (N=207)**

| Variable | Estimate | 95% CI | P-value |
| --- | --- | --- | --- |
| Activity sleep index z-score | -0.39 | -0.78 to -0.001 | 0.049 |
| Education (ref = up to HS) |  |  |  |
| Some college | 0.48 | -0.12 to 1.09 | 0.107 |
| College or above | -0.36 | -1.20 to 0.48 | 0.367 |
| Race and ethnicity (ref = NH White) |  |  |  |
| NH Black | -0.82 | -1.74 to 0.11 | 0.079 |
| Hispanic | -0.45 | -1.03 to 0.13 | 0.115 |
| Others | -0.66 | -1.64 to 0.32 | 0.165 |
| Marital status (ref = married or partnered) |  |  |  |
| Never married | 2.22 | 1.11 to 3.32 | 0.001 |
| Separated or divorced or widowed | 0.43 | -0.18 to 1.05 | 0.150 |
| Age | -0.04 | -0.07 to -0.00 | 0.032 |
| Income level (ref = more than 200% FPL) |  |  |  |
| 100-200% | 0.26 | -0.35 to 0.88 | 0.365 |
| Below FPL | 0.27 | -0.64 to 1.17 | 0.530 |
| History of adverse cardiovascular event (ref = no) |  |  |  |
| Yes | -0.27 | -0.90 to 0.36 | 0.368 |
| Smoked at least 100 cigarettes in lifetime (ref=no) |  |  |  |
| Yes | 0.11 | -0.59 to 0.80 | 0.736 |
| Current insulin use (ref=no) |  |  |  |
| Yes | 1.06 | 0.27 to 1.85 | 0.013 |
| Current oral antihyperglycemic use (ref = no) |  |  |  |
| Yes | 1.26 | 0.38 to 2.15 | 0.009 |
| Current antihypertensive use (ref = no) |  |  |  |
| Yes | -0.23 | -1.56 to 1.10 | 0.709 |
| Current lipid lowering medication use (ref = no) |  |  |  |
| Yes | -0.17 | -1.26 to 0.91 | 0.733 |
